# Misdiagnosis of type 1 and type 2 diabetes in adults

**DOI:** 10.1016/j.lanepe.2023.100661

**Published:** 2023-06-02

**Authors:** The Lancet Regional Health – Europe

Type 1 diabetes, once known as juvenile diabetes, is defined as an autoimmune or idiopathic destruction of β cells that causes severe insulin deficiency in contrast to type 2 diabetes, which is characterised by insulin resistance. Historically, type 1 diabetes has been considered as a disease that primarily affects children and adolescents (aged 10–19 years) and, consequently, diagnosis, clinical care, and advocacy has largely been focused on younger populations. However, findings from recent epidemiological studies and the development of the type 1 diabetes index—a data simulation tool that estimates the number of type 1 diabetes cases for all ages across countries—have shown that the majority of incidence and prevalent cases of type 1 diabetes are in adults. It is estimated that up to 40% of adults older than 30 years with type 1 diabetes might have been misdiagnosed with type 2 diabetes. Considering that the life expectancy of people with type 1 diabetes is reduced by up to 8 years when compared with the general population, in contrast to 3 years for type 2 diabetes, a paradigm shift towards a greater awareness of type 1 diabetes in adults in the clinical and research field is needed. The global agenda, which is overwhelmingly focused on the prevention and treatment of type 2 diabetes in adults, must be expanded to include type 1 diabetes, which has its own challenges, mainly its diagnosis and subsequent appropriate treatment.

One of the fundamental challenges with the misdiagnosis of type 1 diabetes in adults is the assumption that an adult with diabetes would have type 2 diabetes by default. Another important factor contributing to misdiagnosis is that some adults with type 1 diabetes might not need insulin at the time of diagnosis (eg, patients with latent autoimmune diabetes of adults), so their clinical disease might be masked as type 2 diabetes. Additionally, some risk factors for type 2 diabetes, such as obesity and metabolic syndrome, are now much more common in the general population and cannot be used to rule out a diagnosis of type 1 diabetes. Furthermore, quite often there is poor accessibility to perform diagnostic tests, such as islet autoantibodies and C-peptide measurement, that can distinguish type 1 from type 2 diabetes. However, it is also important to acknowledge that the diagnostic algorithm for type 1 diabetes is not as straightforward as for type 2 diabetes, which makes classification even more difficult.

The adverse consequence of type 1 diabetes misdiagnosis is that it affects management of the disease, which is different from type 2 diabetes, and the mismanagement negatively affects the quality of life and survival of patients. The cornerstone treatment of type 1 diabetes is intensive insulin therapy to prevent long-term complications, but treatment strategies should also aim to minimise the psychosocial burden of the disease. Fortunately, the development of new technologies promises to achieve this. Continuous glucose monitoring (CGM), consisting of a subcutaneous sensor that continuously measures glucose levels in interstitial fluid, reduces the number of finger pricks needed, and avoids fluctuations in blood glucose levels thus providing a more personalised care. Regarding insulin delivery, different insulin pumps and hybrid closed-loop systems (also called artificial pancreas or automated insulin delivery) have been shown to be safe and to improve glycaemic control in youths and adults with type 1 diabetes, but require continuous user input. Additional sophisticated technologies are currently being evaluated, such as the bionic pancreas, a fully automated closed-loop system, which is initialised only on the basis of bodyweight without requiring continuous input from the user. Although these advanced technologies are encouraging, their high cost makes them unaffordable for the majority of people with type 1 diabetes.

It is time for the medical, research, and public health community to turn their attention to type 1 diabetes in adults and to develop strategies to tackle the challenges that this disease poses. Health-care professionals should start including type 1 diabetes in their diagnostic arsenal when treating adults, but also the scientific and medical community must push for the development of an effective diagnostic decision tree with specific biomarkers to help correctly classify the type of diabetes. Adults with type 1 diabetes need to be better represented in research and deserve equitable access to novel technologies. A crucial first step is to raise awareness that type 1 diabetes is far more common in adults than previously thought.

